# Cutaneous Nævi Cured by the Application of Iodine Paint

**Published:** 1855-08

**Authors:** S. Edwards

**Affiliations:** Physician-Accoucheur to the Samaritan Hospital for Women, etc.


*Cutaneous Nævi cured by the application of Iodine paint.* By S. Edwards, M. D., Physician-Accoucheur to the Samaritan Hospital for Women, etc.—During the past twelvemonth I have met with two cases of cutaneous nævus in infants, which have been most satisfactorily and completely eradicated by means of the external application of iodine paint.

The first instance in which I was induced to employ it, was for a nævus unfortunately situated on the side of the neck of a female infant. At birth it appeared simply as a small, red shining spot, which in three months increased to the size of a fourpenny piece. The mother of the child at this time positively refusing to have any escharotics employed, fearing that it might give rise to a permanent and greater deformity, I recommended astringent and cold applications to be applied constantly, and this was kept up for some time, but with no good result. The nævus at the end of ten months had acquired additional size, and was observed to become redder and a little more elevated, whenever the circulation was increased by crying, etc. The parents still refusing any of my former suggested remedies, or even of vaccination, "until it got worse," I recommended the use of iodine paint, which was regularly employed by gently painting over the surface with a camel's hair pencil every alternate day, occasionally leaving it off for three or four days when the skin was very irritable and rough. Under this treatment I was pleased to find that the growth of the nævus was arrested, became smaller and mottled, and finally disappeared; a speck or two alone being visible to mark its former site.

The second case was very similar; it occurred in a little boy nearly two years of age. The nævus was about the size of a shilling, but slightly elevated, and situated on the abdomen, and had gradually but very slowly, increased since birth. No treatment had been employed, the Physician who attended the mother of the child, having advised nothing to be done unless it increased. The tincture was commenced in September, 1854, and was continued more or less up to last month when the disease had disappeared, leaving scarce a trace of the mischief.

I know not whether others may have made trial of this treatment, but its success in these two cases has induced me to draw attention to it, as it is a plan so simple in its character that I can see no objection to its employment. In neither of these cases did it produce fever, or, in fact, any effect upon the general health. It is difficult, of course, to decide what might have been the result had these cases been left to themselves without treatment. I have seen several that in the course of time spontaneously disappeared; but still the fact that each of the above cases had gone on increasing up to the commencement of the treatment, and then began shortly to recede, and finally disappeared, must induce the belief that some considerable merit is due to the iodine, and that it deserves a more extended trial.

The many plans that have been proposed and adopted for producing inflammation in, and consequent destruction of, the nævus, are mostly attended with serious objections—*caustics*, by occasional extensive ulcerations, serious hæmorrhages, and by exciting not unfrequently considerable constitutional irritation. *Vaccination* has, I believe generally failed, and when successful has the disadvantage of leaving the ordinary cicatrix. The seton, needles, the injection of fluids, and lastly, and perhaps the best of all, the knife, have an aspect of seriousness to the parents, and are all fraught with occasional serious consequences. The latest plan which has been suggested, is that of Mr. J. B. Brown, who has produced pustules on the cutaneous nævus by means of tartar emetic ointment. Besides the almost certainty of a larger or smaller cicatrix being left, in one of his cases it occasioned very serious slough- of the neighboring parts; objections to its employment about the face and neck of a very decided character.

Under these circumstances, I feel desirous of drawing attention to the above examples, that others may put to the test the value of iodine in these troublesome malformations of the skin, and which, if they would kindly give me the result of their trials, I should esteem it a favor.—*Medical Times and Gazette.*

